# How *ASJ Open Forum* Fits Into Your Aesthetic Education: A Roundtable Discussion

**DOI:** 10.1093/asjof/ojz022

**Published:** 2019-08-12

**Authors:** Jeffrey M Kenkel, Laurie A Casas, Graeme Southwick, Tracy Pfeifer

**Affiliations:** 1 Department of Plastic Surgery, UT Southwestern Medical Center, Dallas, TX; 2 Department of Plastic and Reconstructive Surgery, The University of Chicago Medicine, Chicago, IL; 3 Department of Surgery and Obstetrics and Gynecology, Monash University, Melbourne, Australia

We are pleased to present this roundtable discussion (available online as Supplementary Material at https://academic.oup.com/asjopenforum), recorded live at The Aesthetic Meeting 2019 in New Orleans, featuring a discussion about the newly launched open access publication, *Aesthetic Surgery Journal* (*ASJ*) *Open Forum*. The panelists include Dr. Jeffrey Kenkel, Associate Editor of *ASJ* and *ASJ Open Forum*, Dr. Laurie Casas, Featured Operative Techniques Section Editor, Dr. Graeme Southwick, Editorial Board member, and Dr. Tracy Pfeifer, ASAPS member-at-large. They share their viewpoints on open access publishing and highlight the benefits to potential authors of publication in *ASJ Open Forum.* Key discussion points include a detailed description of the new journal, what article types may be submitted, who can access the published content, and the ease of submission via the cascade process from *ASJ.*

## Disclosures

Dr. Kenkel received research grants from Bellus Medical and Venus; and an educational grant and support for a cadaver course from Galderma. The other authors declared no potential conflicts of interest with respect to the research, authorship, and publication of this article.

## Funding

The authors received no financial support for the research, authorship, and publication of this article.

